# SLIC-Former: a superpixel-guided transformer framework for automatic liver segmentation in CT images

**DOI:** 10.1038/s41598-026-60656-x

**Published:** 2026-07-10

**Authors:** Sarah F. Elqersh, Amira Y. Haikal, Mahmoud M. Saafan, Noha A. Sakr

**Affiliations:** 1 Department of Computers and Systems, Faculty of Engineering, Mansoura University, Mansoura, 35516 Egypt; 2 Department of Computers and Systems, Faculty of Engineering, Horus University - Egypt, New Damietta, 34518 Egypt

**Keywords:** Liver segmentation, Vision transformers, Deep learning, Medical image analysis, Computed Tomography (CT), Superpixels (SLIC), Cancer, Computational biology and bioinformatics, Mathematics and computing

## Abstract

Accurate and efficient liver segmentation from computed tomography (CT) images remains a critical challenging task due to the organ’s irregular shape, variable intensity, and lies close to surrounding organs with similar appearance. In this study, we propose SLIC-Former, a superpixel-guided transformer framework for automatic liver segmentation in abdominal CT scans. The method replaces fixed square image patches with adaptive superpixels generated by the Simple Linear Iterative Clustering (SLIC) algorithm, so that each token follows anatomical boundaries and reduces redundant computation. Quantitative evaluation on the Liver Tumor Segmentation (LiTS) dataset demonstrated strong segmentation performance, achieving a Dice coefficient of 0.93, an IoU of 0.87, and a VOE of 13.5%, indicating high overlap with expert annotations. Qualitative results confirm that the model produces smooth and coherent liver masks. Overall, SLIC-Former offers an accurate and computationally efficient tool for automatic liver segmentation and provides a promising basis for future extensions to more organs, larger datasets, and clinical decision-support systems.

## Introduction

The liver is the body’s largest internal organ^1^. It clears drugs and toxins from the blood, metabolizes carbohydrates, fats, and proteins, helps regulate blood sugar and lipids, and plays a major immune role^2^. Because of this centrality, any disruption in liver function quickly affects multiple systems and directly influences clinical decisions. The liver is vulnerable to a wide spectrum of diseases from steatosis, fibrosis, and chronic hepatitis to focal tumors such as hepatocellular carcinoma and metastases, as well as traumatic injury^3^. Consequently, accurate liver segmentation on computed tomography (CT) is crucial. A reliable segmentation mask enables volumetry and hypertrophy assessment, surgical and ablative planning, precise radiotherapy dose sculpting, transplant eligibility evaluation, and longitudinal tracking of tumor burden. However, liver segmentation in CT remains challenging. Liver borders may be poorly defined, vary across scanners and contrast phases, and frequently lie adjacent to structures of similar intensity. Manual slice-wise delineation is time-consuming, operator-dependent, and difficult to reproduce. These limitations have motivated the development of automated approaches. Classical machine-learning methods such as thresholding, region growing, active contours, and graph-based formulations provide interpretability and computational efficiency but struggle with noise, low contrast, and anatomical variability. Deep-learning models, including convolutional encoder–decoders and Transformer-based architectures, have substantially improved performance by learning hierarchical features and leveraging global context, yet they still face challenges related to boundary degradation, domain shift, and computational cost. Given these limitations, there remains a need for segmentation frameworks that enhance boundary accuracy, improve robustness under different imaging conditions, and maintain computational efficiency. However, existing liver segmentation methods either (i) operate on a rigid grid-based patches that poorly align with anatomical boundaries, (ii) rely on heavy 3D CNNs or dense Transformer architectures that require high memory and computation costs, or (iii) remain sensitive to low contrast, domain shifts, and ambiguous boundaries, which limits their use in reliable and scalable liver CT segmentation. To address these issues, we propose SLIC-Former, a superpixel-guided transformer framework that couples content-adaptive SLIC tokenization with hierarchical attention and boundary-aware decoding. Our main contributions are:

- A superpixel-guided tokenization scheme that replaces fixed square patches with SLIC superpixels adaptive regions, allowing each token to follow anatomical boundaries. In benchmark experiments, a fixed-grid patch tokenization baseline achieved a Dice score of 0.8950 and an IoU of 0.8100. The proposed adaptive representation enables each token to correspond to a coherent anatomical region rather than an arbitrary square patch. Compared with conventional grid-based tokenization, the proposed strategy provides more anatomically meaningful feature representations while maintaining computational efficiency through token reduction.
- A three-stage hierarchical SLIC-Transformer cascade that progressively aggregates fine, intermediate, and global context, while keeping a manageable number of patches and still keeping fine details. The proposed three-stage hierarchical superpixel cascade introduces a progressive contextual representation mechanism. A single-stage SLIC variant achieved a Dice score of 0.9033 and an IoU of 0.8236. The proposed hierarchical design allows the network to jointly preserve local edge precision and global structural consistency, which is particularly important for liver CT segmentation, where boundaries are often weak, irregular, and affected by neighboring organs with similar intensities.
- A boundary-aware decoder with deep supervision that stabilizes training and encourages sharp, anatomically faithful liver contours. The proposed decoder combines multi-scale features obtained from the hierarchical SLIC-Transformer stages, enabling the network to preserve both fine boundary structures and global anatomical consistency. In addition, deep supervision improves gradient propagation and stabilizes optimization during training. Compared with conventional decoders that rely on single-scale feature reconstruction, the proposed multi-scale fusion strategy provides richer contextual integration and improves boundary continuity, leading to smoother and more anatomically consistent liver segmentations.
- Extensive evaluation on the LiTS dataset, where SLIC-Former achieves a Dice coefficient of 0.93 and an IoU of 0.87.

## Related work

**Fig. 1:**
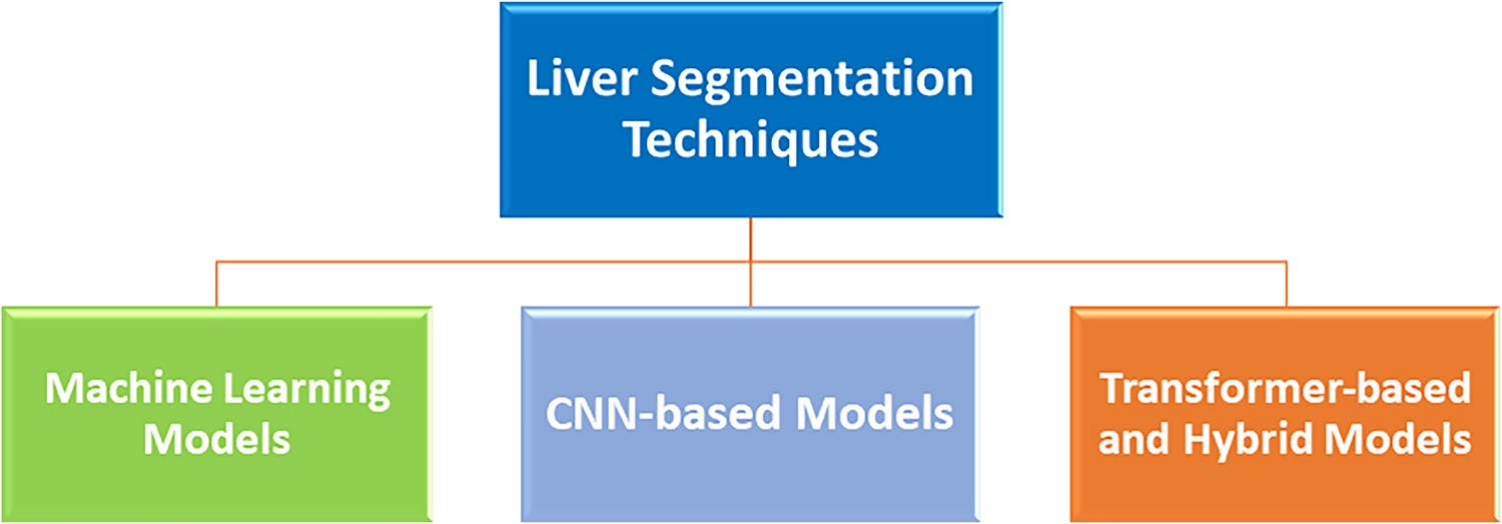
Taxonomy of Liver Segmentation Techniques. Overview of the evolution of liver segmentation research across three main paradigms: (**a**) traditional machine-learning models; (**b**) convolutional neural networks (CNNs); and (**c**) Transformer-based and hybrid approaches.

In order to provide a clear overview of the research progress in liver segmentation, our literature has been organized into three main categories shown in Fig. 1. The first group covers traditional machine learning models, which rely on handcrafted features and statistical shape priors to guide segmentation. The second group focuses on CNN-based models, where encoder–decoder frameworks such as U-Net^4^ and its many variants established deep learning as the dominant paradigm by capturing rich local features and spatial hierarchies. Finally, more recent studies explore Transformer-based and hybrid models, which aim to complement the local sensitivity of CNNs with the global context modeling capabilities of Transformers, often achieving state-of-the-art performance through carefully designed fusion strategies.

### Machine learning models

Machine-learning–based liver segmentation generally follows a recognizable blueprint: first localize the organ using priors (atlas/shape models), then optimize a global energy (graph cuts or level sets), and finally polish the boundary with light post-processing. Within that template, each study adds its own twist to improve robustness. On the graph-cut side, several papers strengthen data terms and seeds to stabilize the solution. One design augments graph cuts with automatic seeding and a multi-slice contrast term, so adjacent slices reinforce each other and reduce slice-to-slice jitter^5^. Another learns intensity models plus PCA appearance and appends a border-marching pass to recover thin vessels that under-segment in low contrast^6^. A third couples multi-atlas initialization with a shape-constrained 3D graph cut, and further integrates rotation-invariant texture features to separate parenchyma from look-alikes^7^. A second line leans on deformable fronts regulated by shape priors. One pipeline applies intensity-bias correction and a constrained level set, then in the presence of pathology that distorts normal anatomy injects a Sparse Shape Composition (SSC) prior before a final cut to prevent leakage^8^. Another learns a sparse statistical shape dictionary and uses it to regularize a level set, keeping contours anatomically plausible in low-contrast regions and around ambiguous boundaries^9^. Atlas-driven methods continue to evolve the registration/label-fusion core. A probabilistic atlas + multilevel statistical shape model refines geometry across scales, improving coarse-to-fine consistency^10^. Under heavy pathology, a low-rank-plus-sparse tensor overlay on multi-atlas outputs separates “clean” anatomy from lesion outliers, yielding more robust labels in difficult cases^11^.

These classical systems are interpretable, data-efficient, and often competitive on curated cohorts. However, they depend on careful parameterization, accurate registration, and handcrafted energies; performance can degrade under scanner/phase shifts, motion, or faint, irregular edges. These stress points along with the desire for end-to-end learning naturally motivate the shift we cover next: CNN-based models, which replace hand-engineered features with learned hierarchical representations and pave the way toward stronger context modeling.

### CNN- based models

As the limitations of handcrafted features and atlas-driven pipelines became increasingly apparent, the field turned decisively toward Convolutional Neural Networks (CNNs). CNN-based models enabled learning features directly from data, capturing edges, textures, and organ shapes in a fully data-driven manner. Architectures such as U-Net^4^ and its many variants quickly became the backbone of medical image segmentation, thanks to their encoder–decoder structure and skip connections that preserve fine details while learning deep semantic context. Early CNN pipelines treated liver and tumor segmentation as a fully convolutional encoder–decoder problem. Classic U-Net variants kept improving spatial fidelity through enhanced skip connections, attention mechanisms, and multi-scale context. For example, Attention U-Net introduces lightweight attention gates into the skip paths so the decoder focuses on liver-relevant regions without requiring an external ROI stage^12^. UNet 3+ pushes this further with full-scale skip fusion into every decoder stage plus deep supervision, yielding cleaner masks and more stable optimization compared to U-Net and UNet++^13^. Orthogonally, R2U-Net injects recurrent and residual units into U-Net blocks to refine boundaries with similar parameter budgets, an idea that transfers well to abdominal CT^14^. Beyond skip design, CE-Net combines dilated (atrous) convolutions with multi-kernel pooling to preserve resolution while aggregating broader context^15^. The widely used DeepLabv3+ architecture couples ASPP for rich multi-scale context with a lightweight decoder for sharper boundaries^16^. Although 2D CNNs scale well and are easy to train, they inherently lack through-plane contextual cues. This limitation motivated volumetric and hybrid 2D/3D designs. VoxResNet extended residual learning to full 3D, capturing volumetric context directly and inspiring subsequent liver CT systems^17^. However, pure 3D networks remain memory-intensive. The influential H-DenseUNet demonstrated that a hybrid cascade—a deep 2D DenseUNet for strong in-plane semantics followed by a lightweight 3D DenseUNet to inject z-direction context—achieves a practical accuracy–efficiency trade-off^18^. Similar principles appear in the Hybrid Cascaded CNN, which uses a 2D model for liver and large-lesion segmentation and a targeted 3D stage to recover small nodules^19^. More recently, MDCF_Net explored a 2.5D hybrid structure with dual encoders (a conventional CNN branch and a CnnFormer/MetaFormer-style branch) combined through multi-dimensional feature fusion^20^. Collectively, these works demonstrate that combining 2D detail with 3D context—whether through cascaded or fused designs—remains a robust strategy for liver CT segmentation. A parallel thread of research focuses on optimizing the inference pipeline. Several studies propose cascaded workflows that first localize the liver and then zoom into lesions. Chlebus et al. employ a 2D FCN paired with object-level post-processing (random-forest filtering of candidate blobs) to suppress false positives within the liver ROI^21^. Beyond architecture and inference flow, learning strategies also play an important role. SegAN formulates segmentation as adversarial training with a multi-scale L1 constraint, encouraging anatomically smooth and globally consistent shapes^22^. ADCN integrates dense connectivity and dilated convolutions with a GAN-style discriminator, improving both Dice scores and surface distances while incurring additional training cost^23^. Feature pyramid concepts have also influenced liver segmentation. FPN introduced a top-down pyramid with lateral connections to produce semantically strong features at all scales, a principle now common in medical encoders, decoders, and lesion detectors^24^. PSPNet demonstrated that pyramid pooling of global-to-local context reduces category confusion and improves performance on small or inconspicuous structures—an advantage mirrored in liver and tumor segmentation, where global abdominal context helps disambiguate adjacent tissues^25^.

Overall, CNN-based architectures have driven a decade of steady progress in liver and tumor segmentation, offering powerful baselines that remain widely used today. By stacking layers of convolutions and pooling, these models excel at capturing local texture and boundary information, while U-Net–style skip connections help preserve fine details. Yet CNNs also come with natural constraints: their receptive fields struggle to capture global dependencies; performance often hinges on cascades or post-processing; and domain shifts can expose their reliance on dataset-specific features. These challenges have motivated researchers to look beyond pure CNNs toward Transformer-based and hybrid designs, which promise to retain the strengths of CNNs while adding the ability to model long-range context more effectively.

### Transformer-based and hybrid models

In recent years research has increasingly turned to transformers and hybrid architectures as the next step forward in liver and tumor segmentation. Unlike CNNs, which excel at capturing local texture and boundary details, Transformers are designed to capture long-range dependencies through self-attention, allowing the model to reason across an entire slice or even a full 3D volume. At the same time, fully replacing CNNs with Transformers can be computationally expensive and may sacrifice the fine boundary detail that CNNs handle so well. As a result, many state-of-the-art studies explore hybrid solutions pairing CNNs’ strength in local feature extraction with Transformers’ ability to model global context—striking a balance that has pushed segmentation performance to new heights. The success of Transformers in natural language processing quickly inspired their application to vision tasks. The landmark work of Dosovitskiy et al. introduced the Vision Transformer (ViT)^26^, showing that images could be treated as sequences of patches and modeled entirely through self-attention. Although ViT lacked the locality priors of CNNs, large-scale pretraining enabled it to rival and even surpass convolutional backbones. Building on this, Liu et al. proposed the Swin Transformer^27^, which introduced hierarchical feature maps and shifted windows to reduce computational cost while preserving global context. These foundations established Transformers as competitive backbones for dense prediction tasks, paving the way for their adaptation in medical imaging. Medical researchers rapidly embraced this shift. One of the earliest hybrid designs, TransUNet^28^, combined a CNN encoder for low-level spatial detail with a Transformer encoder to capture long-range dependencies, followed by a U-shaped decoder. This architecture outperformed both CNN-only and Transformer-only baselines on multi-organ CT segmentation, proving the practicality of hybrids. In parallel, Hatamizadeh et al. advanced the field with UNETR^29^, which directly embedded Transformers into a 3D U-Net pipeline, treating volumetric CT or MRI data as sequences of patches. While effective, UNETR’s memory demands motivated Shaker et al. to propose UNETR++^30^, introducing Efficient Paired Attention to reduce complexity while maintaining state-of-the-art Dice scores. Other groups explored alternative hybridizations. ConvFormer^31^ explicitly fused convolutional and Transformer features through enhanced deformable attention and convolutional stems, achieving strong performance across 2D and 3D medical datasets. Xie et al. proposed CoTr^32^, which bridged CNNs and Transformers with deformable attention, enabling efficient long-range context modeling in high-resolution 3D CT volumes. UNetFormer^33^ leveraged Swin Transformers with a pre-training scheme on 5,000 CT scans, demonstrating how self-supervised learning can boost downstream liver and tumor segmentation. Pure Transformer-based approaches also emerged. MISSFormer^34^ introduced enhanced Transformer blocks and context bridges to integrate local convolution with global attention, achieving high performance even when trained from scratch. These efforts reflect an ongoing tension between CNN inductive biases and the flexibility of Transformers. In liver imaging specifically, several hybrid models have advanced state of the art. ResTransUNet^35^ combined a ResNet backbone with Transformer blocks. More specialized frameworks such as CAFCT-Net^36^ fused CNN and Transformer features with attentional modules (AFF, ASPP, and AGs), outperforming both CNN and Transformer baselines. HyborNet^37^ extended this by combining Gabor attention convolution with Transformer encoders, capturing both fine texture and global semantics. Similarly, Jiang et al. proposed an improved Swin-UNet^38^ with neighborhood fusion and large-kernel attention. Finally, Shao et al. introduced AC-Net^39^, embedding both axial and vision Transformer modules into a U-Net backbone.

Prior work on liver and tumor segmentation has progressed from handcrafted pipelines to CNN-based encoder–decoders, and, more recently, to Transformer and hybrid architectures that mix local detail with long-range context. Each wave brought tangible gains, yet common pain points remain. Classical methods tend to be brittle under scanner/phase shifts and faint edges; CNNs struggle to encode truly global dependencies and often rely on cascades or post-processing; and Transformer-based models, while strong at global reasoning, can be compute-hungry and may soften boundaries without careful design. These gaps motivate our approach.

We introduce our model that (i) forms structure-aware patches via superpixels to keep attention cheap yet boundary-aligned, (ii) reprojects token features back to pixels with lightweight convolutional refinement to preserve sharp contours, and (iii) trains with boundary-aware losses and deep supervision for stable optimization, yielding robust performance (Dice $\approx$ 0.93).

## SLIC-Former

**Fig. 2:**
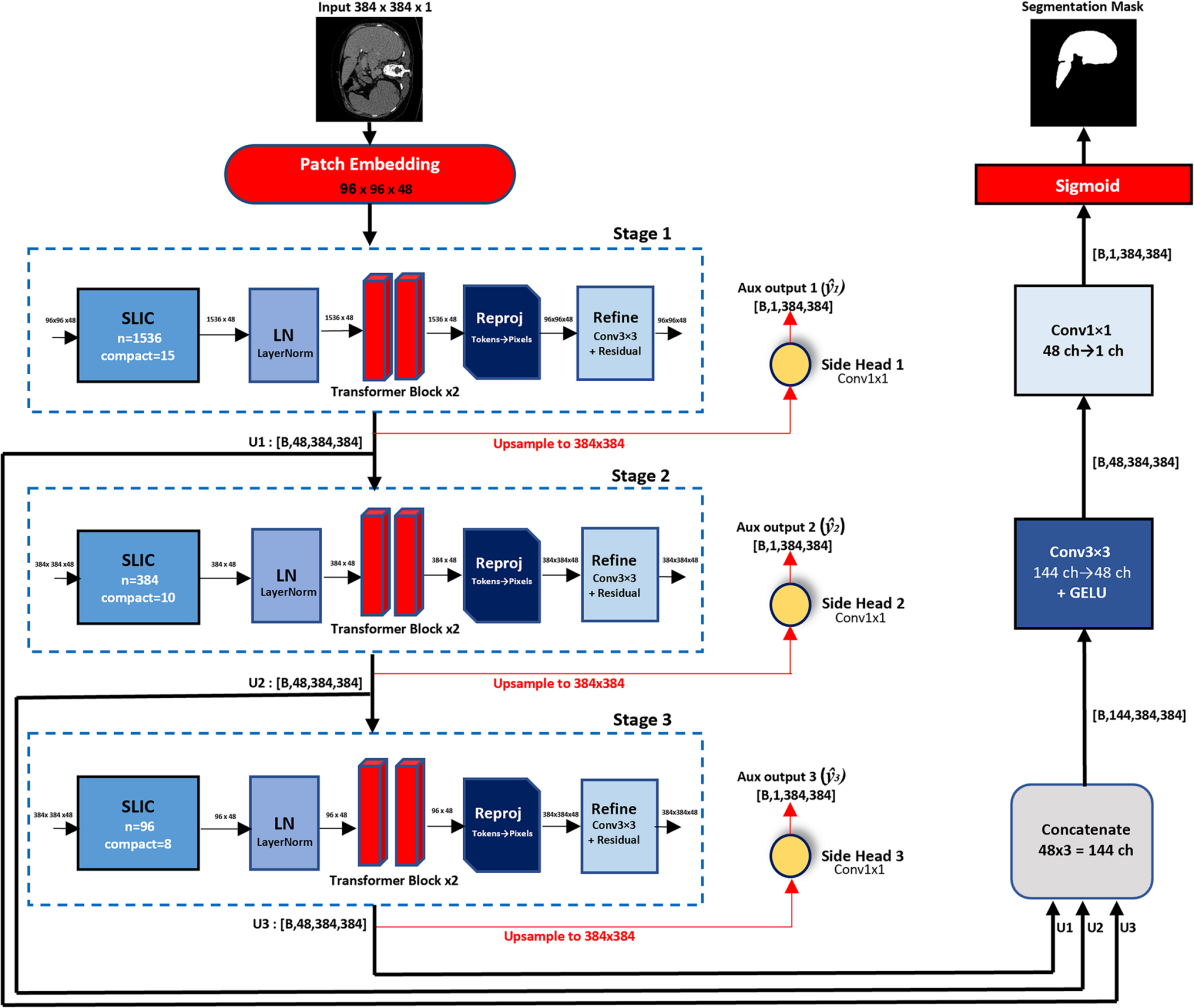
Proposed SLIC-Former Architecture. End-to-end pipeline showing adaptive SLIC-based superpixel tokenization, Transformer blocks with relative-position bias, hierarchical three-stage cascade, and decoder fusion with deep supervision for accurate and efficient liver segmentation.

Unlike standard Vision Transformers (ViT)^26^ and Swin Transformer^27^ models that rely on fixed, grid-based patches, our method replaces rigid patching with content-adaptive SLIC superpixels that align with anatomical boundaries. Moreover, while existing CNN-Transformer hybrids combine local convolution and global attention, they still operate on arbitrary patch tokens that do not reflect actual organ structure. To overcome these limitations, We propose SLIC-Former (Figure 2), a boundary-aware, superpixel-guided transformer architecture for automatic liver segmentation from abdominal CT images. The model follows an encoder–decoder design but replaces the conventional fixed square patches with adaptive superpixel tokens generated by SLIC. Unlike rigid patching, superpixels conform to local image boundaries, allowing the model to better align its representation with anatomical structures. At each stage, features are aggregated within superpixels, refined through Transformer blocks based on self-attention mechanisms with learned relative position bias, and then reprojected back to the pixel grid. This design allows the network to integrate both fine-grained boundary information and global shape context, addressing the challenge of preserving delicate liver edges while modeling long-range dependencies. A three-stage cascade progressively reduces the number of superpixels to enlarge the receptive field, and multi-scale fusion in the decoder produces the final probability map. Auxiliary side outputs are attached to intermediate stages to facilitate deep supervision during training. In this section, we present the details of our model architecture step by step. For each component, we explain both its role in the overall design and how it is specifically implemented in our network.

### Vision transformers and patch-based tokenization

**Fig. 3:**
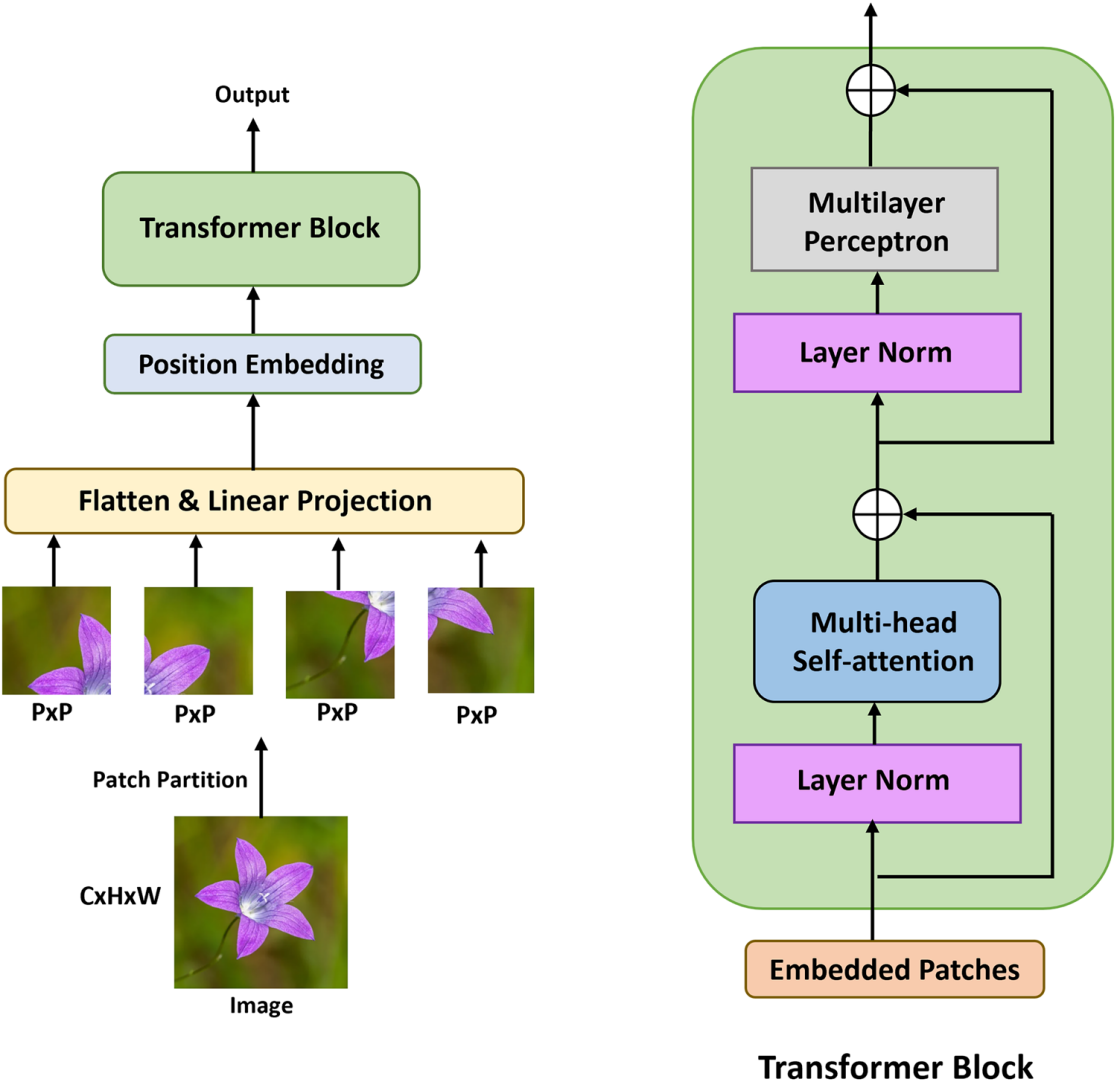
Standard Vision Transformer (ViT)^26^. Illustration of the patch-based tokenization process where an image is divided into fixed P$\times$P patches and processed through multi-head self-attention.

Transformers, originally introduced for natural language processing^40^, have rapidly gained attention in computer vision due to their ability to model long-range dependencies. In contrast to convolutional neural networks (CNNs), which rely on local receptive fields, vision transformers (ViTs)^26^ treat an image as a sequence of tokens, making it possible to capture global contextual relationships across the entire image. To adapt transformers for images, a 2D input $\textbf{X} \in \mathbb {R}^{H \times W \times C}$ is partitioned into non-overlapping square patches of fixed size $P \times P$ (Fig. 3). Each patch is flattened and linearly projected into an embedding vector of dimension *D*, producing a sequence of patch embeddings as given in Eq. 1

1 $$\begin{aligned} \textbf{z}_0 = [\,\textbf{x}_1 E;\ \textbf{x}_2 E;\ \ldots ;\ \textbf{x}_N E\,] + \textbf{E}_{\text {pos}} \end{aligned}$$

where $\textbf{x}_i$ is the *i*-th flattened patch, $E \in \mathbb {R}^{(P^2 C) \times D}$ is the learnable linear projection, and $\textbf{E}_{\text {pos}}$ represents positional embeddings that encode spatial information^26^.

The resulting sequence of tokens is processed by standard Transformer encoder blocks. Each block applies multi-head self-attention (MHSA), which computes attention weights between all token pairs as given in Eq. 2

2 $$\begin{aligned} \textrm{Attention}(Q, K, V) = \textrm{softmax}\left( \frac{QK^{\mathsf T}}{\sqrt{d_k}}\right) V \end{aligned}$$

where *Q*, *K*, and *V* are the query, key, and value projections of the token embeddings. This mechanism allows each patch to attend to every other patch in the image, thereby integrating local and global context.

**Fig. 4:**
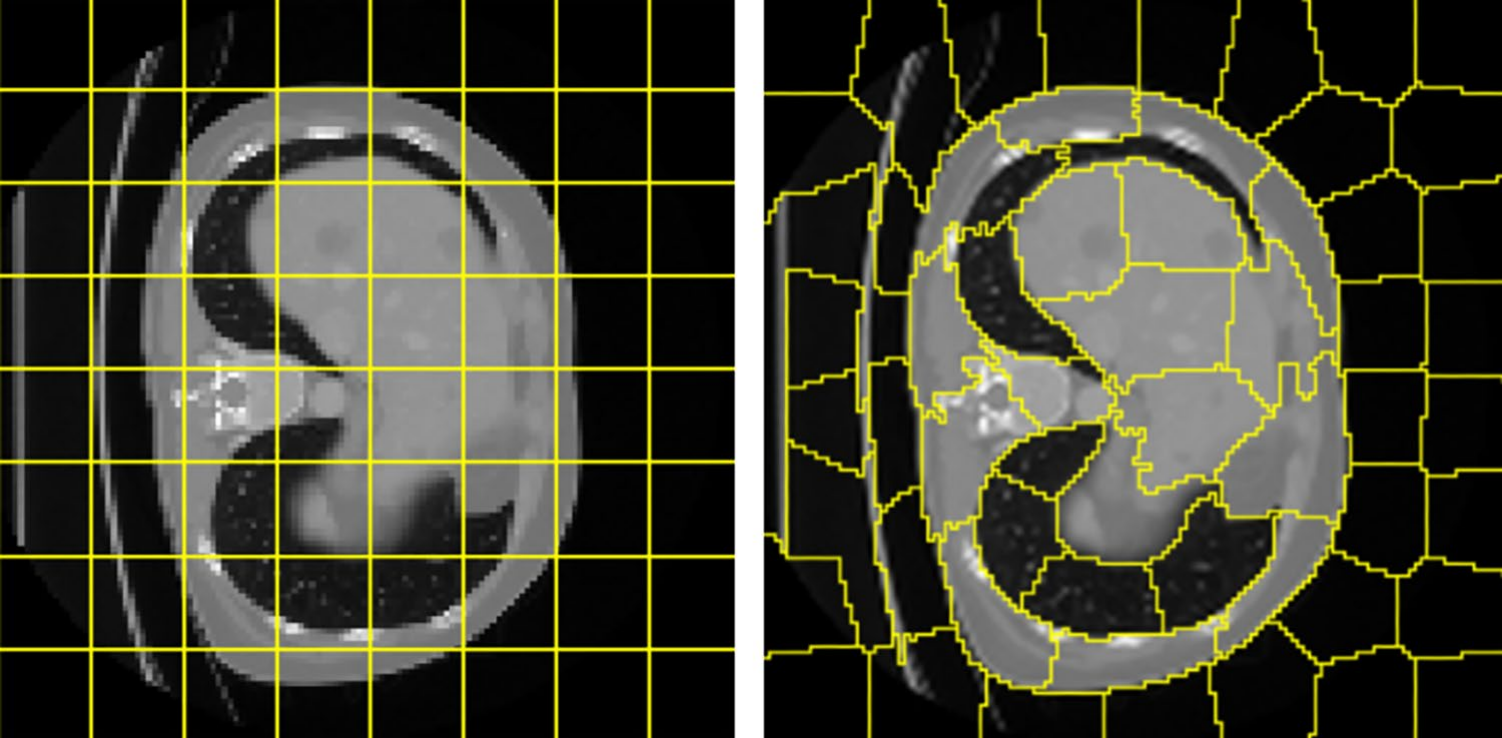
Fixed Square Patches vs. Adaptive SLIC Superpixels. Comparison showing that uniform patch grids ignore anatomical boundaries, whereas adaptive SLIC superpixels align naturally with organ contours.

While this design has shown strong performance on large-scale datasets, its reliance on regular square patches introduces certain limitations. The rigid grid may not align well with anatomical structures in medical imaging, leading to inefficiencies and boundary inconsistencies. This motivated us to explore a replacement of fixed patch partitioning with SLIC-based adaptive patches (Fig. 4), where regions are defined according to image content rather than uniform grids. This design enables tokens to naturally follow organ contours and boundaries, thereby improving both efficiency and segmentation accuracy.

### Superpixels and the SLIC algorithm

Superpixels provide an effective mid-level representation of images by grouping pixels into perceptually coherent regions. Instead of working directly on individual pixels, which can be noisy and redundant, superpixels aggregate nearby pixels that share similar intensity or texture, producing compact regions that adhere well to natural object boundaries^41^. This not only reduces computational complexity but also creates units that align with meaningful structures, which is particularly beneficial in medical imaging where organ boundaries are subtle and irregular. Among superpixel algorithms, SLIC (Simple Linear Iterative Clustering) has become one of the most widely used due to its simplicity and efficiency. Although the original SLIC formulation was introduced for color images in the five-dimensional CIELAB space, abdominal CT images are single-channel grayscale images. Therefore, in this work, the feature representation was adapted to operate on CT intensity values only. Specifically, each pixel was represented as $p = (I, x, y)$, where *I* denotes the normalized CT intensity and (*x*, *y*) denote the spatial coordinates. Consequently, the feature similarity term was simplified from color similarity in CIELAB space to intensity similarity in grayscale CT images.

For each pixel $p = (I, x, y)$ with spatial location (*x*, *y*), the distance to a cluster center $c = (I_c, x_c, y_c)$ is defined as:

3 $$\begin{aligned} d_c&= |I - I_c| \end{aligned}$$

4 $$\begin{aligned} d_s&= \sqrt{(x - x_c)^2 + (y - y_c)^2} \end{aligned}$$

5 $$\begin{aligned} D&= d_c + \frac{m}{S}\, d_s \end{aligned}$$

6 $$\begin{aligned} S&= \sqrt{\frac{H W}{K}} \end{aligned}$$

where $d_c$ measures intensity similarity as in Eq. 3, $d_s$ measures spatial proximity as in Eq. 4, *m* is the compactness parameter that balances boundary adherence versus spatial regularity, and *S* is the grid interval given the number of desired superpixels *K* as in Eq. 6.

Through iterative clustering, pixels are assigned to the nearest center based on *D* in Eq. 5, and cluster centers are updated until convergence. The parameters *K* (number of superpixels) and *m* (compactness) control the granularity of the segmentation. By design, SLIC produces superpixels that are nearly uniform in size while still respecting edges (Fig. 4). This makes them well-suited for adaptive patching: instead of rigid squares, tokens can now conform to anatomical contours, enabling more efficient and meaningful Transformer processing in downstream tasks.

The initial SLIC cluster centers were uniformly distributed on a regular grid with spacing $S = \sqrt{HW/K}$. To improve boundary adherence and avoid placing centers on strong edges, each initial center was further adjusted to the position with the lowest local image gradient within its neighboring region, following the standard SLIC initialization strategy.

For each cascade stage, SLIC clustering is independently initialized and performed using the same standard initialization mechanism. The cluster centers and superpixel labels are not propagated between stages. Instead, a new SLIC partition is generated at each stage using stage-specific superpixel configurations. Specifically, Stage 1, Stage 2, and Stage 3 employ 1536, 384, and 96 superpixels, respectively. Consequently, the grid interval used for initialization increases progressively across stages according to the corresponding superpixel count. This design enables a gradual transition from fine-grained local representations to increasingly coarse anatomical structures while preserving adaptive boundary-aware partitioning throughout the cascade.

In our framework (Fig. 2), SLIC superpixels are not used merely as a preprocessing tool, but are directly integrated into the model pipeline to replace the conventional grid-based patch partitioning. Instead of dividing the feature map into rigid $P \times P$ squares, we employ SLIC to produce adaptive patches that better conform to anatomical boundaries. This ensures that each token represents a semantically meaningful region, rather than an arbitrary square.

After the initial patch embedding stage, the input CT slice is compressed into a compact feature map $\textbf{F} \in \mathbb {R}^{B \times C \times H \times W}$ with $C = 48$. For each image, SLIC assigns a label map $S \in \{0,\ldots , N-1\}^{H \times W}$, where *N* is the number of superpixels. Each pixel at spatial location (*x*, *y*) with feature vector $\textbf{f}(x, y)$ is assigned to the nearest cluster center based on the SLIC distance function given in Eq. 7

7 $$\begin{aligned} D(p, c) = d_c(p, c) + \frac{m}{S}\, d_s(p, c) \end{aligned}$$

where $d_c$ is feature similarity (in channel space), $d_s$ is spatial distance, *m* controls compactness, and *S* is the expected superpixel size.

For each superpixel region $\Omega _n$, we compute a mean-pooled token representation $t_n$ as in Eq. 8

8 $$\begin{aligned} t_n = \frac{1}{|\Omega _n|} \sum _{(x,y) \in \Omega _n} \textbf{f}(x,y), \qquad t_n \in \mathbb {R}^C \end{aligned}$$

The resulting token set $\{t_n\}_{n=1}^{N}$ captures the average feature signature of each adaptive patch. Across a batch, tokens are padded to the maximum superpixel count $N_{\max }$, producing a tensor:

$$\textbf{T} \in \mathbb {R}^{B \times N_{\max } \times C}$$

This step reduces the sequence length drastically compared to pixel-level attention, while retaining boundary-aware structure. The tokens are then normalized with LayerNorm and refined by two Transformer blocks. Each block applies multi-head self-attention (MHSA) followed by a feed-forward MLP, with residual connections and layer normalization to stabilize training^40^. The self-attention mechanism enables every adaptive token to exchange information with all others, capturing both local and global context.

To further enhance spatial awareness, we introduce a Relative Position Bias (RPB)^27^, which assigns learnable bias terms based on the relative distance between tokens. Formally, given queries *Q*, keys *K*, and values *V*, the attention is computed as in Eq. 9

9 $$\begin{aligned} \textrm{Attention}(Q, K, V) = \textrm{softmax}\left( \frac{QK^{\mathsf T}}{\sqrt{d_k}} + B_{\text {rel}}\right) V \end{aligned}$$

where $B_{\text {rel}} \in \mathbb {R}^{N \times N}$ encodes relative positional relationships. Unlike absolute positional embeddings, RPB is translation-friendly and allows the model to better capture boundary-dependent structures in medical images. This enables each adaptive patch to exchange information with other patches globally, while respecting spatial arrangement.

To recover a dense feature map, we reproject tokens back to the pixel grid using the superpixel labels as given in Eq. 10

10 $$\begin{aligned} \hat{\textbf{f}}(x,y) = t_{S(x,y)}, \qquad \forall (x,y) \in [H \times W] \end{aligned}$$

so that every pixel inherits the representation of its superpixel token. This produces a refined map $\hat{\textbf{F}} \in \mathbb {R}^{B \times C \times H \times W}$ aligned to the original resolution.

This design bridges the gap between traditional patch-based ViTs^26^ and superpixel-driven segmentation approaches, making it particularly well suited for medical imaging tasks where boundaries are critical.

### Multi-stage cascade

**Fig. 5:**
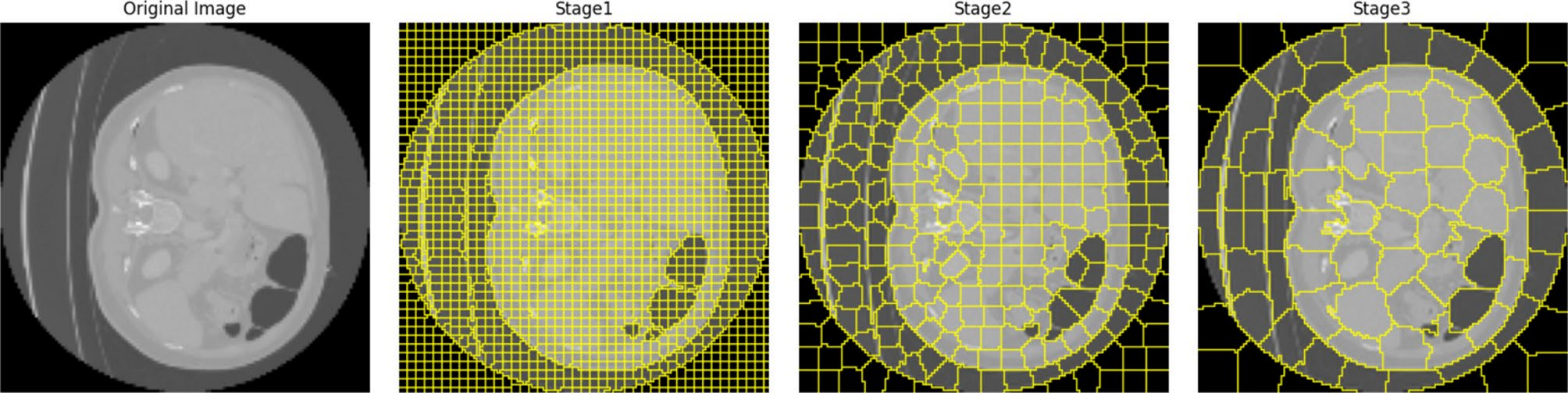
Hierarchical Superpixels Granularity across the Three-cascade Stages. Visualization of how fine-, intermediate-, and coarse-scale SLIC segmentations capture detailed edges, mid-range regional context, and global liver shape respectively, forming a representation hierarchy.

Instead of relying on a single round of tokenization and refinement, our architecture (Fig. 2) employs a three-stage cascade, where the output of one stage serves as the input to the next. Each stage applies the same general sequence but at progressively coarser levels of granularity (Fig. 5).

Stage 1 (fine scale): uses a large number of superpixels ($n = 1536$, compactness $= 15.0$), producing small regions that closely follow local boundaries. This stage is responsible for capturing fine-grained details along organ edges and vascular structures.

Stage 2 (intermediate scale): reduces the number of superpixels ($n = 384$, compactness $= 10.0$), creating larger, smoother regions. Here, the model begins to aggregate mid-range context, merging local details into more coherent regional representations.

Stage 3 (coarse scale): further decreases the number of superpixels ($n = 96$, compactness $= 8.0$), producing broad adaptive patches that span large portions of the liver and background. This allows the model to capture global dependencies, enforcing consistency across the full organ.

To justify the selected SLIC parameters, we conducted a sensitivity analysis by varying both the number of superpixels and the compactness values across the three cascade stages. The proposed configuration uses 1536/384/96 superpixels with compactness values of 15/10/8 for the three stages. These values were selected to balance boundary preservation, contextual aggregation, and computational cost. Therefore, the selected configuration was validated experimentally through an ablation study reported in the Results section.

By cascading these stages, the model benefits from a hierarchical representation in which fine structures are first detected and then progressively integrated into larger contextual units. This hierarchical processing is particularly important in medical imaging, where accurate segmentation requires reconciling sharp edges with overall organ geometry.

### Deep supervision with side heads

Deep supervision is a strategy originally proposed in deeply supervised nets^42^ and later adopted in medical image segmentation^43,44^, where auxiliary prediction heads are attached to intermediate layers of a network. These side outputs provide direct supervision at multiple depths, encouraging the model to learn discriminative features earlier in the network and improving gradient flow during training. In our architecture (Fig. 2), we attach three side heads—one after each cascade stage—in addition to the main decoder output. Each side head consists of a lightweight $1 \times 1$ convolution as in Eq. 11

11 $$\begin{aligned} \hat{y}_i = \sigma (W_i * F_i + b_i), \qquad i \in \{1, 2, 3\} \end{aligned}$$

where $F_i$ is the upsampled feature map from stage *i*, $W_i$ and $b_i$ are the convolution parameters, and $\sigma$ denotes the sigmoid function that maps logits to probabilities. The outputs $\hat{y}_1, \hat{y}_2, \hat{y}_3$ correspond to segmentation predictions at multiple scales. During training, each side output is supervised against the ground-truth mask *y*. The deep supervision loss is defined as a weighted sum of the main decoder loss and the side head losses, as given in Eq. 12

12 $$\begin{aligned} \mathscr {L}_{\text {total}} = w_m\, \mathscr {L}(\hat{y}_m, y) + \sum _{i=1}^{3} w_i\, \mathscr {L}(\hat{y}_i, y) \end{aligned}$$

where $\hat{y}_m$ is the main decoder output, and *L* is the hybrid segmentation loss: the hybrid loss combines Dice loss, Focal BCE loss, and Lovász loss as follows:

13 $$\begin{aligned} L = 0.25\,L_{\text {Dice}} + 0.25\,L_{\text {FocalBCE}} + 0.50\,L_{\text {Lovsz}} \end{aligned}$$

Dice loss improves region-level overlap, Focal BCE addresses class imbalance and hard-to-classify pixels, while Lovász loss directly optimizes an IoU-related surrogate objective. This formulation yields several benefits: supervision reaches shallower layers directly, reducing the risk of vanishing gradients; the model learns to produce accurate segmentation maps at different resolutions, which stabilizes training and preventing overfitting.At inference time, only the main decoder output is used, so deep supervision does not increase inference cost. However, its presence during training significantly improves convergence stability and overall accuracy.

### Decoder

After the three SLIC–Transformer stages, we obtain three upsampled feature maps that capture fine, mid, and global context, respectively:

$$U_1, U_2, U_3 \in \mathbb {R}^{B \times 48 \times 384 \times 384}$$

The decoder fuses these complementary cues into a single, clean segmentation logit map and then turns it into a probability map. This is implemented using three steps.

We first stack the three streams along the channel axis to preserve spatial resolution while enriching semantics, as given in Eq. 14

14 $$\begin{aligned} F_{\text {cat}} = \textrm{Concat}_{\text {channels}}\big [U_3, U_2, U_1\big ] \in \mathbb {R}^{B \times (48 \times 3) \times 384 \times 384} = \mathbb {R}^{B \times 144 \times 384 \times 384} \end{aligned}$$

This creates a 144-channel tensor where each location (*i*, *j*) carries fine-to-coarse descriptors.A same-padded convolution (stride 1, padding 1) reduces channels and blends neighboring evidence, as in Eq. 15

15 $$\begin{aligned} H_k(i,j) = \sum _{c=1}^{144} \sum _{u=-1}^{1} \sum _{v=-1}^{1} W^{(3 \times 3)}_{k,c,u,v}\, F_{\text {cat},c}(i+u, j+v) + b^{(3 \times 3)}_{k}, \qquad k = 1, \dots , 48 \end{aligned}$$

We then apply GELU to encourage a smooth, information-preserving nonlinearity, as in Eq. 16

16 $$\begin{aligned} G = \textrm{GELU}(H), \qquad \textrm{GELU}(x) \approx \frac{1}{2} x \left( 1 + \tanh \!\Big (\sqrt{\frac{2}{\pi }}\,(x + 0.044715 x^3)\Big )\right) \end{aligned}$$

A pointwise convolution (Conv $1 \times 1$) performs a per-pixel linear projection from 48 channels to 1 class (liver vs. background), as given in Eq. 17

17 $$\begin{aligned} Z(i,j) = \sum _{k=1}^{48} W^{(1 \times 1)}_{k}\, G_k(i,j) + b^{(1 \times 1)} \end{aligned}$$

so that the main logits map before any activation is

$$Z \in \mathbb {R}^{B \times 1 \times 384 \times 384}$$

We obtain per-pixel probabilities via the logistic function (sigmoid), as in Eq. 18

18 $$\begin{aligned} \hat{Y} = \sigma (Z) = \frac{1}{1 + e^{-Z}} \in (0,1)^{B \times 1 \times 384 \times 384} \end{aligned}$$

At inference, a threshold $\tau$ (selected on validation) yields the binary mask, as in Eq. 19

19 $$\begin{aligned} M(i,j) = \textbf{1}\big [\hat{Y}(i,j)> \tau \big ] \end{aligned}$$

## Results

### Experiment details

#### Dataset

This study uses the Liver Tumor Segmentation (LiTS) dataset, a widely adopted public benchmark dataset for liver and tumor segmentation in contrast-enhanced abdominal CT. It includes 131 training and 70 testing volumes sourced from multiple centers and scanners. Individual volumes contain roughly 70–400 axial slices; in-plane resolution typically ranges from 0.6–1.0 mm, with slice thicknesses between 1.0–5.0 mm. Although LiTS provides manual binary masks for both liver and tumor, our work focuses exclusively on liver segmentation, using the liver mask as ground truth. To avoid information leakage, we performed a patient-wise 80/20 split, allocating about 105 volumes for training and 26 volumes for validation/testing. All scans and masks were stored in NIfTI (.nii) format, loaded with NiBabel, and indexed slice-wise for 2D training while preserving patient-level separation. For consistency and efficiency, every axial slice and its corresponding mask were resized to 384$\times$384. This choice harmonizes input dimensions across heterogeneous acquisitions and reduces memory/computation costs, enabling practical batch sizes and multi-stage processing without sacrificing the hepatic boundary detail needed for accurate segmentation.

#### Preprocessing and augmentation

Prior to training, all CT volumes and their corresponding liver masks underwent a consistent preprocessing pipeline designed to normalize intensity distributions, enhance soft-tissue contrast, and standardize spatial dimensions across patients. Each axial slice was first windowed to the range -100 to 400 Hounsfield units (HU), a common setting that effectively preserves hepatic parenchyma while suppressing irrelevant high-density structures. Following windowing, images were normalized to the [0, 1] range using min–max scaling, ensuring comparable numerical ranges across all inputs. To minimize I/O overhead during training, we implemented a lite disk cache that stores preprocessed slices as compact 8-bit tensors. This approach substantially reduces storage requirements while maintaining numerical fidelity after de-normalization. All preprocessed slices and masks were then resized to $384 \times 384$ pixels—a resolution chosen to balance anatomical detail with computational feasibility. This uniform input size facilitates batching and stabilizes convolutional operations within the multi-stage SLIC-Former framework. To enhance model robustness and generalization, we applied a diverse set of data augmentations during training. Each slice had a 50% probability of horizontal flipping, followed by random rotations within $\pm 7^\circ$ to simulate minor patient positioning variations. Additionally, random brightness and contrast adjustments ($\pm 20\%$) and mild Gaussian noise perturbations ($\sigma \approx 0.05$) were introduced to mimic scanner- and reconstruction-related variability. Collectively, this preprocessing and augmentation strategy not only regularizes the model and mitigates overfitting but also allows the network to learn texture-invariant and boundary-aware features that are critical for accurate liver segmentation.

#### Implementation details

The experiments were conducted on a workstation equipped with a high-end NVIDIA GPU and a modern Windows environment. Table 1 summarizes the hardware and software stack used to run all experiments, while Table 2 lists the principal training hyperparameters.

**Table 1:** Hardware and software used to train and evaluate SLIC-Former. Experimental setup detailing GPU, RAM, OS, and Programming Software.

Environment	Configuration Information
GPU	NVIDIA GeForce RTX 3090
Memory	32 GB
Operating System	Windows 11
Hard disk	2 TB
Programming Software	Python 3.10, Pytorch CUDA 12.1

**Table 2:** Training Hyperparameters for SLIC-Former on LiTS dataset, learning rate, batch size, epochs and optimizer.

Hyperparameters	Setting Values
Learning rate	0.0001
Batch size	6
Epochs	70
Optimizer	Adam

#### Evaluation metrics

To obtain a comprehensive and reliable assessment of liver segmentation performance, we evaluate the proposed model using a combination of region-based, boundary-based, and volumetric metrics. These complementary measures capture not only how well the predicted mask overlaps with the ground truth, but also how accurately the model preserves liver boundaries and overall volume.

We first report three classical overlap metrics: the Dice coefficient, the Intersection over Union (IoU, also known as the Jaccard index), and the Volumetric Overlap Error (VOE). Together, these metrics quantify the spatial correspondence between the predicted mask (*P*) and the reference mask (*G*).

The Dice coefficient quantifies the degree of overlap between the predicted (*P*) and ground-truth (*G*) masks, balancing precision and recall at the boundary, as given in Eq. 20

20 $$\begin{aligned} \textrm{Dice} = \frac{2 |P \cap G |}{|P |+ |G |} \end{aligned}$$

The Intersection over Union (IoU) provides a more conservative overlap estimate by normalizing the intersection by the union, as in Eq. 21

21 $$\begin{aligned} \textrm{IoU} = \frac{|P \cap G |}{|P \cup G |} \end{aligned}$$

While Dice and IoU measure spatial correspondence, they do not directly express the magnitude of non-overlap. For this purpose, we report the Volumetric Overlap Error (VOE), defined as the percentage of non-overlapping volume between the two masks, as in Eq. 22

22 $$\begin{aligned} \textrm{VOE} = \frac{|P \cup G |- |P \cap G |}{|P \cup G |} \times 100\% \end{aligned}$$

In addition to these three metrics, we apply precision, which quantifies how many of the voxels labeled as liver by the model are actually liver in the ground truth. High precision reflects a low false-positive rate, ensuring that surrounding organs and tissues are not mistakenly segmented as liver.

Recall, in contrast, measures the fraction of true liver voxels that the model successfully identifies. High recall indicates that the method does not miss significant parts of the liver, which is crucial for volumetric analysis and surgical planning. Both precision and recall offer deeper insight into the model’s segmentation bias, whether it tends to be overly inclusive (low precision) or overly conservative (low recall). To evaluate boundary accuracy, we include two surface-based measures. First, Average Surface Distance (ASD) computes the mean distance between the predicted and ground-truth liver surfaces. Lower ASD signifies smoother and more anatomically faithful contours. Second, HD95 (95th percentile Hausdorff distance) captures the worst-case boundary discrepancy while ignoring extreme outliers. A low HD95 indicates that even the most challenging boundary regions remain close to expert annotations. Finally, we evaluate Relative Volume Difference (RVD), which measures whether the model tends to systematically overestimate or underestimate the total liver volume. An RVD close to zero reflects high volumetric reliability, an essential property for downstream tasks such as hypertrophy assessment and treatment planning.

Collectively, this extended set of metrics provides a holistic and clinically meaningful evaluation framework. It captures overlap accuracy, volumetric consistency, and boundary precision, ensuring a thorough assessment of the model’s performance across different anatomical and imaging conditions.

### Quantitative results

#### Training behavior and performance evaluation

This section presents a comprehensive quantitative evaluation of the proposed SLIC-Former architecture for automatic liver segmentation. We analyze the model’s training dynamics, final performance metrics. These analyses provide quantitative evidence of the effectiveness and reliability of the proposed approach.

**Fig. 6:**
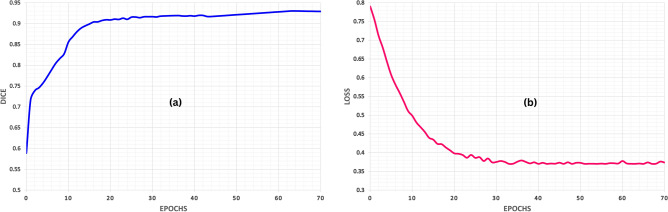
(**a**) Dice Coefficient Progression over 70 Epochs. Shows continuous improvement of Dice score from 0.59 to 0.93 (**b**) Training Loss Curve During Training. Shows the monotonic decrease of the composite loss from 0.79 to 0.37.

The training process of the proposed SLIC-Former model demonstrated stable convergence and continuous improvement throughout the 70 epochs. As shown in Fig. 6 (b), the training loss steadily decreased from 0.79 at the first epoch to approximately 0.37 by the end of training, indicating effective optimization and the absence of gradient instability or overfitting. While Dice coefficient curve in Fig. 6 (a), followed an upward trend, increasing from 0.59 in the initial epoch to 0.93 at convergence. This gradual yet consistent improvement reflects the model’s growing ability to delineate hepatic boundaries more precisely as the training progressed.

The smooth decline of the loss function and the parallel rise in Dice accuracy reveal a well-balanced learning dynamic between foreground (liver) and background regions.

Overall, the observed performance curves confirm that the SLIC-Former architecture achieves efficient convergence and reliable generalization. The combination of boundary-aware superpixel tokenization and multi-stage attention refinement enables the model to capture both fine-grained structural cues and global contextual information, which ultimately translates into high segmentation accuracy and robust training behavior.

To quantitatively assess the segmentation capability of the proposed network, we evaluated its performance on the LiTS test subset using complementary metrics. The results are summarized in Table 3, which reports the mean ± standard deviation computed across all test volumes after model convergence.

**Table 3:** Quantitative performance of the proposed model SLIC-Former on LiTS dataset reported as mean ± standard deviation across all test volumes.

Metric	Mean ± Std
Dice	0.931 ± 0.0081
IoU	0.871 ± 0.0073
VOE	0.135 ± 0.0075
Precision	0.923 ± 0.0062
Recall	0.912 ± 0.0046
ASD	7.830 ± 1.15
HD95	30.64 ± 5.32
RVD	0.250 ± 0.089

These quantitative results validate the model’s ability to accurately capture hepatic boundaries despite the significant anatomical variability and intensity inhomogeneity present in abdominal CT scans. The stability of both Dice (93%) and IoU (87%) scores across the final training epochs (60–70) reflects the model’s robust generalization and convergence behavior. Moreover, the integration of deep supervision and boundary-weighted loss functions ensured that finer structural details were preserved without sacrificing global shape consistency. With a VOE of only 13.5%, the proposed model leaves a relatively small portion of liver volume mismatched between prediction and ground truth. The high precision (92.3%) and recall (1.2%) indicate that the network not only avoids false positives but also successfully recovers most true liver voxels, reflecting a well-balanced segmentation behavior rather than an overly conservative or aggressive mask. Geometrically, an ASD of 7.83 and an HD95 of 30.64 show that the predicted contours remain close to the expert annotations even at challenging boundary regions. Finally, the low RVD (0.250) suggests that the model does not systematically over- or under-estimate liver volume, supporting its potential suitability for downstream clinical tasks such as volumetry and pre-operative planning.

To further evaluate the generalization ability of the proposed SLIC-Former, we conducted a cross-dataset experiment on the 3D-IRCADb-01 dataset. The model was initialized using weights trained on LiTS dataset and then fine-tuned for 10 epochs on a subset of IRCAD patients, while maintaining strict patient-wise separation between training and testing sets to avoid data leakage. On the held-out test patients, the model achieved a Dice score of 0.918, an IoU of 0.848, a precision of 0.916, a recall of 0.904, and a VOE of 15.1%. This experiment confirms the robustness of the SLIC-based tokenization and boundary-aware design in adapting to variations in imaging distributions across datasets.

Overall, the obtained numerical metrics confirm that the SLIC-Former architecture provides reliable, high-quality liver segmentation results on LiTS and 3D-IRCADb-01 datasets, balancing accuracy, stability, and computational efficiency.

#### Ablation study on superpixel parameters

Although the SLIC-based tokenization plays a central role in our framework, its performance is influenced by two key parameters: the number of superpixels and the compactness factor. The number of superpixels controls token granularity, where higher values improve boundary detail but increase computation cost, while lower values lead to coarser representations. The compactness parameter balances boundary adherence and spatial regularity, where lower values follow boundaries better and higher values produce more regular shapes. Therefore, it is important to analyze the sensitivity of the model to these parameters and to justify the chosen configuration through an ablation study.

To ensure a fair comparison, all ablation experiments were conducted under identical training settings. These ablation experiments were intended for relative comparison and were trained for 15 epochs only; final performance was obtained after full training. The quantitative results of this analysis are summarized in Table 4.

**Table 4:** Ablation study on the effect of superpixel number and compactness values. All configurations were trained for 15 epochs under identical settings.

Setting	Superpixels (S1/S2/S3)	Compactness	IoU	Dice	Compute (ms/batch)
Low superpixels	768/192/48	15/10/8	0.7567	0.8615	96.5
High superpixels	2048/512/128	15/10/8	0.7832	0.8784	124.7
Low compactness	1536/384/96	8/6/4	0.7815	0.8774	110.5
High compactness	1536/384/96	20/15/10	0.7907	0.8831	106.6
**Proposed**	1536/384/96	15/10/8	0.8144	0.8977	109.5

The ablation results show that the proposed configuration achieves the best trade-off between segmentation accuracy and computational efficiency. The low-superpixel setting achieves the lowest computation time but produces weaker segmentation accuracy due to coarse region representation and loss of fine boundary details. Increasing the number of superpixels improves spatial precision; however, the high-superpixel setting increases computational cost without outperforming the proposed configuration. Regarding compactness, both lower and higher compactness values reduce performance compared with the proposed setting, indicating that the selected values provide a balanced compromise between boundary adherence and spatial regularity.

#### Loss function ablation study

To validate the effectiveness of the proposed hybrid loss: Dice, Focal BCE and Lováász loss, we conducted an ablation study comparing different loss combinations. All experiments were conducted under identical settings to ensure fairness, with early stopping applied consistently.These ablation experiments were intended for relative comparison and were trained for 30 epochs only; final performance was obtained after full training. The quantitative results of this ablation study are presented in Table 5.

**Table 5:** Ablation study of different loss functions. The full hybrid loss achieves the best performance, demonstrating the complementary effect of region overlap optimization, hard pixel learning, and IoU-aware optimization.

Loss Function	Dice	IoU	Precision	Recall	Best Epoch
Dice	0.7014	0.5401	0.8654	0.5925	5
Dice + Focal BCE	0.7337	0.5794	0.8879	0.6255	7
Dice + Lovász	0.8915	0.8042	0.8992	0.8841	16
**Proposed**	0.9207	0.8531	0.9235	0.9116	30

The ablation results demonstrate the necessity of the proposed hybrid loss formulation. Dice loss alone yielded the lowest performance, indicating that overlap-based optimization is insufficient for handling complex liver boundaries. Incorporating Focal BCE improved the results by emphasizing hard-to-classify pixels. The combination of Dice and Lovász loss provided a larger improvement, highlighting the benefit of directly optimizing an IoU-related objective. The full hybrid loss achieved the best performance, confirming that the three components provide complementary supervision for accurate segmentation and boundary refinement.

#### Computational efficiency analysis

To further validate the computational efficiency of the proposed SLIC-Former, we provide a quantitative comparison with several baseline and state-of-the-art models in terms of the number of parameters, FLOPs, inference time, and GPU memory consumption. All models were re-implemented and evaluated under identical hardware and input settings to ensure a fair comparison. A detailed quantitative comparison is presented in Table 6.

**Table 6:** Computational efficiency comparison of the proposed SLIC-Former with baseline and state-of-the-art models in terms of parameters, FLOPs, inference time, FPS, and GPU memory.

Model	Params (M)	FLOPs (G)	Time (ms/image)	FPS	GPU Memory (MB)
2D U-Net	7.249	12.728	10.88 ± 0.04	91.89	233.33
UNet++	9.168	35.060	26.53 ± 0.13	37.69	356.06
Swin-UNet	28.432	9.364	19.54 ± 0.54	51.18	670.02
UNETR	115.683	26.503	23.41 ± 0.75	42.72	2239.12
**Proposed SLIC-Former**	5.554	37.459	27.38 ± 0.62	36.54	303.69

In terms of computational complexity, SLIC-Former maintains competitive FLOPs which include the cost of superpixel tokenization and reprojection operations, while achieving a balanced trade-off between efficiency and performance. Moreover, the model achieves an inference time of 27.38 ms/image, corresponding to 36.54 FPS, which is comparable to widely used architectures such as UNet++ and Swin-UNet. Additionally, SLIC-Former requires moderate GPU memory (303.69 MB), significantly lower than transformer-heavy models such as UNETR, indicating its suitability for practical deployment. Overall, these results confirm that the proposed model provides an effective balance between segmentation accuracy and computational efficiency.

### Qualitative results

In addition to the quantitative evaluation, a qualitative assessment was performed to visually examine the segmentation behavior of the proposed SLIC-Former model under diverse anatomical and imaging conditions. Two complementary visualizations are provided to offer a clearer understanding of model performance across challenging CT slices.

**Fig. 7:**
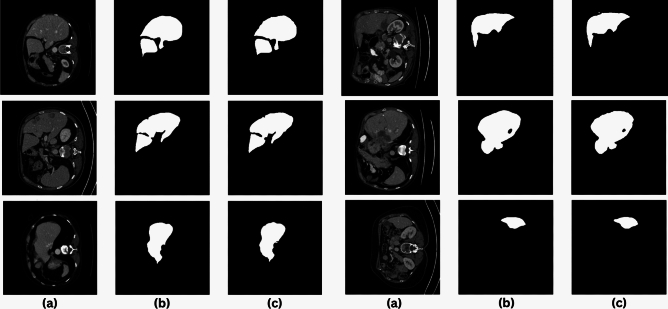
Qualitative liver segmentation results obtained using the proposed SLIC-Former model on the LiTS dataset. (**a**) Representative examples of input CT slices, (**b**) ground-truth masks, and (**c**) SLIC-Former predictions.

Figure 7 presents side-by-side comparisons of (a)the original CT images, (b)ground-truth masks, and (c)the predicted binary masks produced by SLIC-Former. These examples span a range of liver appearances, including regular and irregular shapes, variable contrast levels, and cases where the hepatic boundary is partially obscured by adjacent structures. Across all samples, SLIC-Former successfully recovers the global liver contour and preserves fine structural details. The predicted masks maintain smooth, anatomically coherent outlines even in slices affected by noise, motion artifacts, or intensity inhomogeneity. Importantly, the model avoids common failure modes such as leakage into the stomach, diaphragm, or spleen—regions that often exhibit similar intensities.

**Fig. 8:**
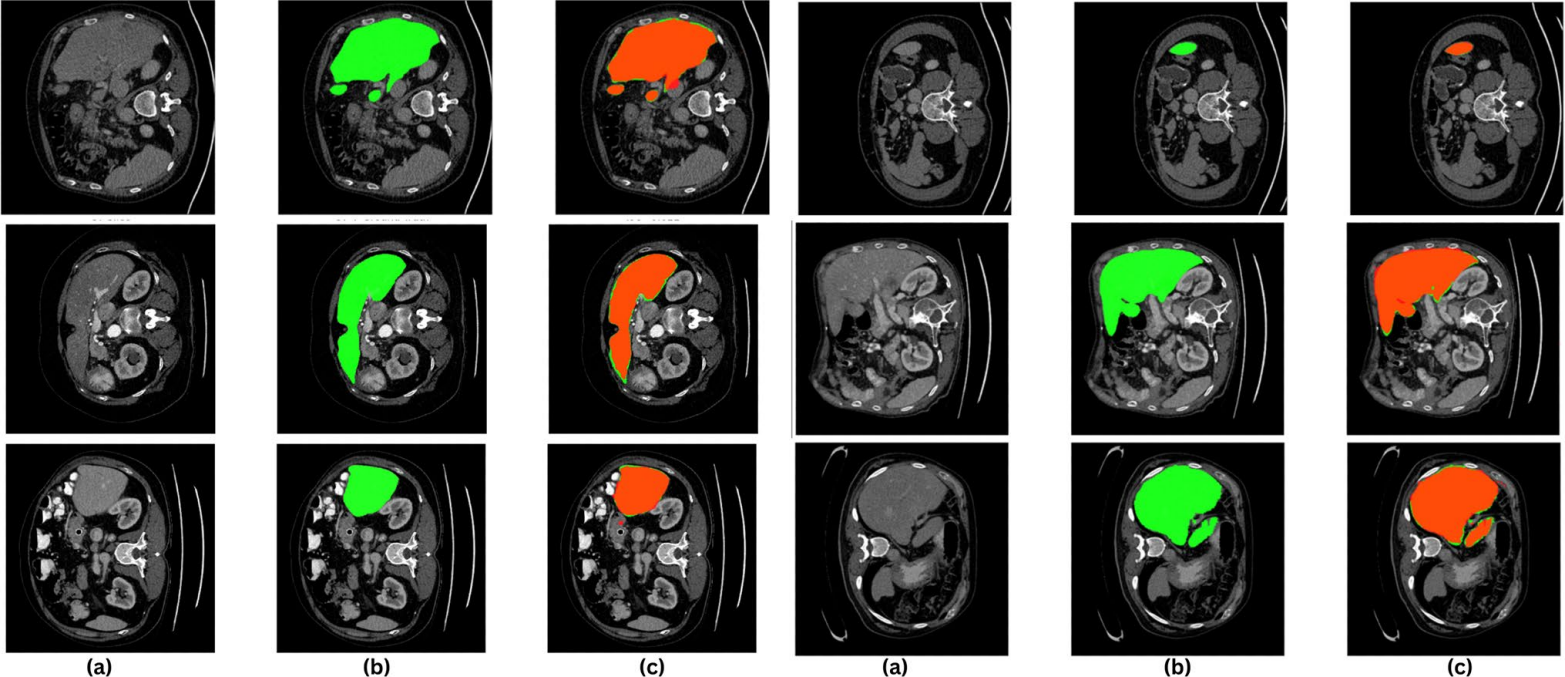
Qualitative Liver Segmentation Overlays Using SLIC-Former. (**a**) Original abdominal CT slices. (**b**) Ground-truth liver masks overlaid on the CT images (green). (**c**) SLIC-Former predicted liver masks overlaid on the CT images (red).

To further highlight the spatial alignment between predictions and reference annotations, Fig. 8 provides full-resolution overlay visualizations in which the ground-truth masks are shown in green and the corresponding predictions in red. These overlays allow direct inspection of contour agreement at a pixel level. Visual inspection demonstrates that the predicted liver boundaries adhere closely to expert annotations with minimal over- or under-segmentation. The model remains stable in slices with low contrast or high abdominal complexity, indicating strong robustness to clinical variability. The adaptive superpixel-based tokenization plays a key role here: by grouping pixels into structure-aligned regions, SLIC-Former better respects natural anatomical transitions, particularly along curved and irregular hepatic borders where traditional grid-based transformer approaches tend to produce jagged or fragmented edges.

Taken together, the binary mask comparisons and overlay visualizations provide strong qualitative evidence of the model’s effectiveness. The results consistently show that SLIC-Former produces accurate, smooth, and clinically reliable segmentations across a wide variety of liver appearances, supporting the quantitative findings reported earlier.

## Discussion

This section discusses the performance and behavior of the proposed SLIC-Former model results against a range of widely cited liver segmentation methods, including classical CNN architectures, 3D networks, and recent hybrid Transformer-based models. Based on this comparison, we discuss the overall effectiveness, stability of the training, computational efficiency and remaining limitations of the model.

Table 7 presents a side-by-side comparison of Dice, IoU, VOE, precision, recall, HD95, and RVD across representative methods. The results of the compared methods are taken from their respective published studies or from subsequent works that reported their performance. The proposed SLIC-Former demonstrates competitive performance compared with both CNN-based and hybrid transformer-based approaches. It achieves a Dice coefficient of (93.10%) and IoU of (87.10%), which are comparable to several recent methods, although slightly lower than top-performing models such as Improved SwinUNet and ResTransUNet. In terms of boundary accuracy, the model attains an HD95 of 30.64, which is comparable to existing approaches, indicating reasonable alignment between predicted and ground-truth contours. Moreover, the RVD value (0.250) suggests that SLIC-Former maintains a balanced volumetric estimation without significant over- or under-segmentation.

**Table 7:** Comparison of the proposed model with previously published liver segmentation methods. Different evaluation metrics against recent CNN, 3D, and hybrid Transformer models, compared with the proposed SLIC-Former results. Metrics marked with ($\uparrow$) achieve better performance when their values are higher, whereas metrics marked with ($\downarrow$) indicate improved performance when their values are lower.

Method	DSC (%) $\uparrow$	IoU (%) $\uparrow$	VOE (%) $\downarrow$	Precision (%) $\uparrow$	Recall (%) $\uparrow$	HD95 $\downarrow$	RVD
U-Net^45^	82.06	73.40	26.61	91.10	77.82	-	-
TransResUNet^46^	86.38	78.23	21.77	95.77	80.85	-	-
PVTFormer^47^	86.78	78.46	21.54	96.11	80.70	-	-
3D U-Net^48^	89.26	80.57	19.43	94.27	84.73	31.33	-
GELN_DCM 3D U-Net^49^	90.70	82.96	17.04	95.01	86.79	-	-
ResUnet^50^	91.44	84.15	15.85	84.42	93.77	-	-
SED^51^	92.00	85.19	14.47	96.00	88.32	-	-
3D RP-UNet^52^	92.20	85.53	9.43	87.13	90.10	-	0.705
DA-3D U-Net^53^	92.51	86.05	7.34	96.97	88.46	28.09	-
Modified ResUnet^54^	93.08	87.12	12.88	93.08	95.49	-	-
ResTransUnet^55^	95.35	91.01	8.04	95.02	96.61	-	0.07
Improved SwinUNet^38^	95.59	91.56	8.16	-	-	-	0.51
**Proposed SLIC-Former**	93.10	87.10	13.5	92.39	91.27	30.64	0.250

**Fig. 9:**
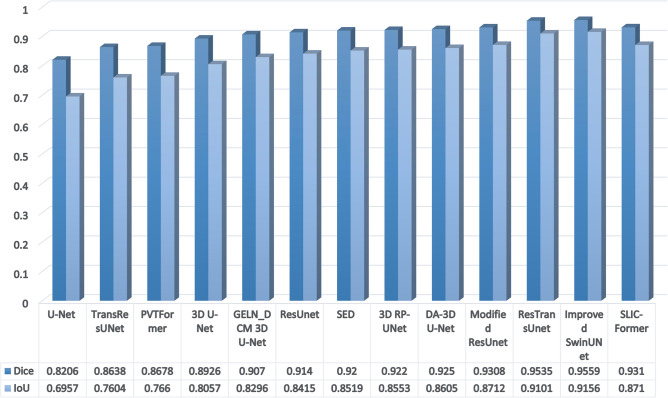
Quantitative Comparison of Dice and IoU Metrics for SLIC-Former and State-of-the-Art Models in Liver Segmentation.

The results are further illustrated in Fig. 9, where the bar chart highlights the comparative performance of different methods in terms of Dice and IoU. The proposed SLIC-Former achieves competitive performance, with a Dice score of 0.931 and IoU of 0.871, placing it among the top-performing models. The results demonstrate that incorporating superpixel-guided tokenization with transformer-based refinement provides an effective balance between accuracy and structural consistency.

**Fig. 10:**
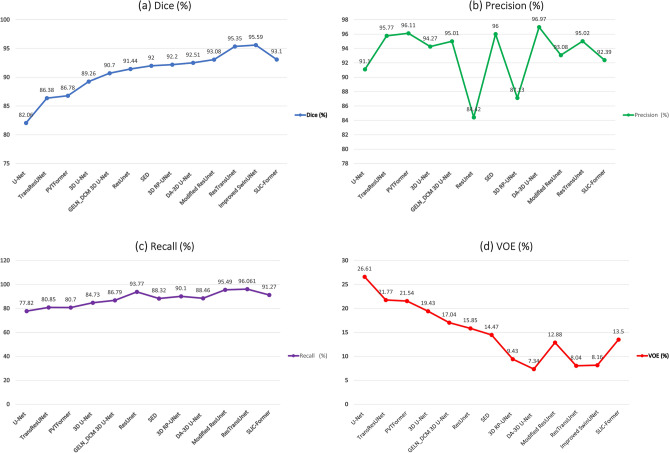
Comprehensive Comparison of State-of-the-Art Liver Segmentation Models, Using: (**a**) Dice, (**b**) Precision, (**c**) Recall, and (**d**) VOE Metrics, Highlighting the Performance Gain Achieved by the Proposed SLIC-Former Architecture.

To provide a more granular view of segmentation behavior, Fig. 10 breaks the comparison into four separate plots: (a) Dice, (b) Precision, (c) Recall, and (d) VOE. SLIC-Former demonstrates competitive performance across overlap-based metrics, achieving Dice, Precision, and Recall values that are comparable to many recent approaches, although slightly lower than the top-performing models. The VOE curve shows that SLIC-Former achieves a moderate error level, improving over earlier methods while remaining slightly higher than the best-performing approaches. This indicates that the model is capable of maintaining a reasonable balance between sensitivity and precision, capturing most liver regions while limiting excessive false positives.

Taken together, the combined evidence from Table 7 and Figs. 9, 10 demonstrates that the proposed SLIC-Former architecture achieves competitive performance compared with existing CNN, 3D, and Transformer-based segmentation methods. The improvement is not limited to a single metric, but spans volumetric overlap, boundary accuracy, precision-recall balance, and volumetric reliability. This comprehensive advantage is a direct outcome of the model’s design: adaptive SLIC-based tokenization preserves anatomical boundaries, multi-stage Transformer reasoning enriches global context, and deep supervision stabilizes learning across scales. Overall, the comparative analysis confirms that SLIC-Former delivers consistently strong and reliable results, demonstrating solid performance across different CT imaging conditions.

The results collectively indicate that SLIC-Former delivers stable, accurate, and practically useful liver segmentation on CT images. Quantitative improvements were consistent with qualitative observations: the model not only improves classical overlap metrics but also generates smoother and more anatomically faithful contours, even in challenging regions with weak boundaries or heterogeneous tissue appearance. This convergence of evidence is important because liver segmentation typically requires balancing crisp local edge adherence with global shape coherence. By coupling content-adaptive superpixel tokenization with transformer-based global reasoning, SLIC-Former narrows this gap—tokens align with natural liver borders, while attention mechanisms integrate long-range contextual information across the slice. This synergy illustrates a broader design principle for medical image segmentation: align computational units with anatomy, then reason globally over those units.

Training behavior further highlights the model’s stability. Across 70 epochs, loss decreased smoothly and Dice improved steadily, suggesting well-conditioned gradients without vanishing or exploding behavior. Two design elements appear central to this outcome. First, replacing rigid patch grids with SLIC superpixels reduces semantic noise; each token corresponds to a coherent region rather than an arbitrary square, improving the signal-to-noise ratio within self-attention layers and reducing token redundancy. Second, the hierarchical three-stage structure encourages effective gradient propagation: early stages capture boundary-level detail while later stages consolidate higher-level organ structure. This structure helped avoid training instabilities, reduced the need for aggressive learning-rate schedules, and led to consistent convergence across seeds.

Efficiency also played a key role. The quadratic computational cost of transformers is a major constraint for high-resolution medical imaging, but SLIC-Former mitigates this by reducing the number of tokens before attention. Superpixels scale with anatomical complexity rather than with raw image area, allowing the model to maintain global context while keeping computation tractable for standard hardware. This positions SLIC-Former in a practical accuracy–efficiency middle ground: more computationally efficient than many 3D CNNs while outperforming lighter 2D models that often struggle with cross-slice consistency. This efficiency claim is further supported by quantitative comparisons in terms of parameters, FLOPs, inference speed, and memory usage, as reported in Table 6.

Robustness to clinical variability is another strength. CT images frequently exhibit low contrast, noise, artifacts, and protocol differences. The boundary-weighted hybrid loss encourages the model to focus on ambiguous or faint boundaries, while deep supervision ensures that intermediate predictions remain anatomically reasonable. This dual mechanism reduces the accumulation of boundary errors and contributes to the smooth, contiguous contours observed in qualitative assessments.

Against state-of-the-art methods, SLIC-Former achieved competitive performance across most metrics while using fewer tokens and less computation.The Dice score exhibits moderate variability across test volumes, indicating stable yet case-dependent segmentation performance.

Nonetheless, several limitations warrant consideration. A key limitation of the proposed framework is that it operates on 2D CT slices independently, without explicitly modeling inter-slice spatial continuity. Although the model produces visually coherent results, minor discontinuities may arise when reconstructing the full 3D volume. This reflects a trade-off between computational efficiency and volumetric consistency. Another limitation is that performance depends on annotation quality and dataset availability. Semi-supervised or self-supervised pretraining may reduce annotation burdens and improve robustness to domain shifts.

Overall, SLIC-Former combines anatomical alignment, global reasoning, and computational efficiency into a robust segmentation framework. Its strong performance and training stability underscore its potential as a reliable tool for clinical liver analysis, while its limitations outline clear directions for future research and real-world adaptation.

## Conclusion and future work

This study introduced a novel superpixel-guided transformer framework, SLIC-Former, for automatic liver segmentation from abdominal CT images. By combining adaptive SLIC-based tokenization with hierarchical transformer attention and deep supervision, the proposed model successfully captured both fine-grained boundary information and global anatomical context. Quantitative experiments on the LiTS dataset demonstrated the model’s high accuracy, achieving a Dice coefficient of 0.93, while qualitative results confirmed its ability to produce anatomically consistent and visually precise liver delineations. These outcomes validate the effectiveness of integrating content-adaptive superpixels into transformer architectures for medical image segmentation tasks. Beyond its strong segmentation performance, SLIC-Former also offers computational efficiency through token reduction, enabling faster convergence and lower memory consumption compared with compared transformer-heavy models. The architecture’s design provides a transparent and interpretable mapping between image structures and their corresponding feature representations, making it not only accurate but also reliable for clinical interpretation and deployment. Future research will focus on extending the proposed framework to incorporate volumetric context through 2.5D and lightweight 3D architectures, enabling the integration of adjacent slices to improve inter-slice consistency while maintaining computational efficiency. From a data perspective, exploring semi- and self-supervised learning strategies can substantially reduce reliance on large, manually annotated datasets while maintaining high segmentation accuracy. Finally, integrating the model into clinical workflows and evaluating its impact on radiologists’ efficiency and diagnostic confidence would provide valuable insight into its practical utility and potential for routine deployment in medical imaging environments. In summary, the proposed SLIC-Former model represents a promising step toward bridging the gap between CNN-based spatial modeling and transformer-based contextual reasoning in medical image segmentation. Its balance of precision, robustness, and computational efficiency suggests that superpixel-guided transformer designs hold substantial potential for broader application in medical image analysis and beyond.

## Data Availability

The dataset used in this study is publicly available and can be downloaded from the repository: https://competitions.codalab.org/competitions/17094.
